# The diagnostic value of quantitative bone SPECT/CT in solitary undetermined bone lesions

**DOI:** 10.3389/fonc.2023.1205379

**Published:** 2023-11-09

**Authors:** Fen Du, Xieraili Wumener, Yarong Zhang, Ming Liu, Taichuang Li, Size Huang, Maoqun Zhang, Rongliang Wu, Ying Liang

**Affiliations:** Department of Nuclear Medicine, National Cancer Center/National Clinical Research Center for Cancer/Cancer Hospital and Shenzhen Hospital, Chinese Academy of Medical Sciences and Peking Union Medical College, Shenzhen, China

**Keywords:** bone lesions, undetermined bone lesions, quantitative single-photon emission computed tomography, standardized uptake value, Tc-99m methylene-diphosphonate

## Abstract

**Objective:**

To investigate the diagnostic value of the maximum standard uptake value (SUVmax) of quantitative single-photon emission computed tomography/computed tomography (SPECT/CT) in solitary undetermined bone lesions.

**Methods:**

In Part I, retrospective study, 167 untreated patients with extra-skeletal malignant tumors by pathology were consecutively enrolled for staging with Tc-99m methyl-diphosphonate (^99m^Tc-MDP) whole-body bone scan (WBS) and quantitative SPECT/CT, and a total of 396 bone lesions with abnormal radioactivity concentration in 167 patients were included from April 2019 to September 2020. The differences in SUVmax among the benign bone lesions, malignant bone lesions, and normal vertebrae were analyzed. The receiver operating characteristic (ROC) curve and cutoff value of SUVmax were obtained. Part II, prospective study, 49 solitary undetermined bone lesions in SPECT/CT in 49 untreated patients with extra-skeletal malignant tumors were enrolled from October 2020 to August 2022. The diagnostic efficacy of SUVmax in solitary undetermined bone lesions was assessed. The final diagnosis was based on follow-up imaging (CT, MRI, or 2-deoxy-2-[18F]fluoro-D-glucose-positron emission tomography/computed tomography) for at least 12 months.

**Results:**

In Part I, a total of 156 malignant and 240 benign bone lesions was determined; the SUVmax of malignant lesions (26.49 ± 12.63) was significantly higher than those of benign lesions (13.92 ± 7.16) and normal vertebrae (6.97 ± 1.52) (*P* = 0.00). The diagnostic efficiency of the SUVmax of quantitative SPECT/CT revealed a sensitivity of 75.00% and a specificity of 81.70% at a cutoff value of 18.07. In Part II, 17 malignant and 32 benign lesions were determined. Using SUVmax ≥18.07 as a diagnostic criterion of malignancy, it has a sensitivity of 82.35%, a specificity of 93.75%, and an accuracy of 89.80%.

**Conclusion:**

The SUVmax of quantitative SPECT/CT is valuable in evaluating solitary undetermined bone lesions. Using a cutoff SUVmax value of 18.07, quantitative SPECT/CT demonstrated high sensitivity, specificity, and accuracy in differentiating malignant from benign bone lesions.

## 1 Introduction

Bone is the third most common site of metastasis for malignant tumors after lung and liver, especially in osteophilic tumors, such as prostate, lung, breast, and thyroid cancer (1). It has been reported that the relative incidence of bone metastases varies between tumors, 65%–75% in prostate cancer, 65%–75% in breast cancer, 30%–40% in lung cancer, 40% in bladder cancer, 20%–25% in renal cell carcinoma, and 14%–45% in melanoma (2). Early identification of bone metastases in patients with malignant tumors is essential for staging and appropriate treatment selection. Whole-body bone scan (WBS) is one of the most commonly used methods to identify bone metastases early (3). Although WBS reveals a high sensitivity to detect bone lesions, it has specificity and spatial resolution limitations. With the addition of single-photon emission computed tomography (SPECT) and single-photon emission computed tomography/computed tomography (SPECT/CT) to WBS, the specificity limitations have been gradually improved. For instance, 62% of the lesions undetermined by WBS could be identified by SPECT (4) and 85%–92% of the lesions undetermined by SPECT could be identified by SPECT/CT (5). However, there are still difficulties in making a definitive diagnosis for some bone lesions in SPECT/CT (such as SPECT-positive/CT occult lesions, that is, focal abnormal radioactivity concentration on SPECT images in the absence of an identifiable anatomic lesion on CT images, or SPECT-positive/CT positive lesions, that is, focal abnormal radioactivity concentration on SPECT images with an identifiable anatomic lesion on CT images, but the diagnosis cannot be confirmed), especially in solitary lesions, which require further examination and are more expensive (6).

Quantitative analysis, widely used in positron emission tomography/computed tomography (PET/CT), has unique advantages such as objectivity, accuracy, and reproducibility compared with traditional qualitative analysis (7). In 2020, Zeintl et al. found that current commercially available SPECT/CT techniques can perform ^99m^Tc SPECT quantitative imaging with reasonable accuracy in both model (error <3.6%) and patient (error <1.1%) studies (8). Furthermore, with the improvement of SPECT/CT in terms of attenuation correction, scatter correction, and detection performance in recent years, SPECT/CT quantitative analysis is gradually being used in clinical practice. The study performed by Arvola et al. showed strong correlations between the SUV values measured for ^18^F-NaF PET/CT and ^99m^Tc HDP SPECT/CT, thus indicating the feasibility of using SPECT/CT SUVs in clinical practice (9). Yamane et al. also demonstrated that SUVmax of quantitative SPECT/CT has good repeatability and could be a reliable indicator in patient management (10).

Although the accuracy of SUVmax might be affected by equipment hardware, acquisition conditions, and reconstruction methods (11–13), many clinical studies have demonstrated the potential to serve as a promising biomarker of osteoblastic metabolism of SUVmax of the quantification bone SPECT/CT (14–17). Zhang et al.’s (14), Tabotta et al.’s (16), and Kuji et al.’s (17) studies already showed that the SUVmax of bone quantitative SPECT/CT is helpful to the differential diagnosis of benign and malignant bone lesions in prostate, breast, lung, liver, and thyroid cancer patients. Based on previous studies, our study attempts to determine the cutoff value of SUVmax to the differential diagnosis of benign and malignant bone lesions through retrospective analysis and then prospectively investigate the diagnostic value of the SUVmax in the evaluation of solitary undetermined bone lesions.

## 2 Materials and methods

### 2.1 Patients

#### 2.1.1 Part I retrospective study

A total of 256 untreated patients with extra-skeletal malignant tumors by pathology were consecutively retrospectively enrolled for staging with Tc-99m methyl-diphosphonate (^99m^Tc-MDP) whole-body bone scan (WBS) and quantitative SPECT/CT from April 2019 to September 2020. The main excluded criteria were (i) patients lost to follow-up or (ii) follow-up for less than 12 months. Finally, 167 patients were enrolled (104 men and 63 women, mean age 59.76 ± 11.46 years, range 22–86 years), including 98 with lung cancer, 16 with nasopharyngeal carcinoma, 11 with esophageal cancer, 9 with breast cancer, 9 with malignant uterine tumor, 6 with prostate cancer, 4 with rectal cancer, 3 with thyroid cancer, 3 with renal cancer, 2 with hypopharyngeal cancer, 2 with oral cancer, 1 with bladder cancer, 1 with gastric cancer, 1 with lacrimal cancer, and 1 with squamous carcinoma of the tympanic ventricle.

#### 2.1.2 Part II prospective study

A total of 49 untreated patients with extra-skeletal malignant tumors by pathology and a solitary abnormal radioactivity concentration undetermined in conventional WBS and SPECT/CT were prospectively analyzed from October 2020 to August 2022. The exclusion criteria are consistent with Part I. Among these patients, 35 men and 14 women (mean age 59.57 ± 12.15 years, range 30–84 years), 32 with lung cancer, 8 with esophageal cancer, 2 with malignant uterine tumor, 2 with rectal cancer, 2 with renal cancer, 1 with nasopharyngeal carcinoma, 1 with breast cancer, and 1 with malignant melanoma.

In Part I and Part II, all patients were followed up for at least 12 months (20.71 ± 5.32, 12.00–38.65 months). For ethical and practical reasons, biopsy-based confirmation of patient bone metastases was not performed; instead, the gold standard for diagnosis was based on follow-up imaging, such as CT, MRI, or 2-deoxy-2-[18F]fluoro-D-glucose (^18^F-FDG)-positron emission tomography (PET)/CT. The Ethics Committee of the National Cancer Center/National Clinical Research Center for Cancer/Cancer Hospital & Shenzhen Hospital, Chinese Academy of Medical Sciences and Peking Union Medical College approved the present study (JS2023-15) and waived the requirement for patient-informed consent.

### 2.2 Image acquisition and processing

The anterior and posterior whole-body scan was performed using a SPECT/CT scanner (GE Discovery NM/CT 670 Pro, United States) equipped with a low-energy high-resolution collimator at 160–280 (average 193.11 ± 23.76) minutes after intravenous injection of 684.50–937.50 (average 887.63 ± 76.59) MBq ^99m^Tc MDP (HTA Co., Ltd., Guangzhou). Subsequently, based on the findings of abnormal radioactivity concentration from the WBS, the patients underwent regional quantitative SPECT/CT scanning. SPECT images were initially acquired with an energy peak of 140 keV, a 10% window (126–154 keV), and step-and-shoot mode acquisition (10 s per step, 30 steps per detector) with a 6° angular increment. Scattering correction is mainly achieved by setting an extra window at 120 keV with a 5% window (114–126 keV). Next, CT imaging was performed in the same position, using the following parameters: tube voltage, 120 kV; tube current, 150 mA; table feed, 27.5 mm per rotation; tube rotation time, 0.8 s; pitch, 1.375:1; and matrix, 512 × 512. CT images thus obtained were reconstructed into 3.75-mm-thick sections using an adaptive statistical iterative reconstruction algorithm (ASiR; GE Healthcare). For SPECT, images were reconstructed using an iterative ordered subset expectation maximization algorithm (2 iterations, 10 subsets) with CT-based attenuation correction, scatter correction, and resolution recovery using the software package provided by the vendor (Volumetrix Mi; GE Healthcare). A post-reconstruction filter was also applied (Butterworth filter; frequency of 0.48, order of 10). After reconstruction, images were set on a 128 × 128 matrix with a 3.75-mm section thickness and a 1.0 zoom factor.

Regular calibration of the SPECT/CT system was performed with an internal ^57^Co point source phantom. The SPECT-reconstructed values were decay-corrected to the time of injection, and final values of quantitative radioactivity concentration were obtained to allow maximum SUV body weight quantification (SUVmax) on post-processed images. The delineation of the volume of interest (VOI) was performed using the quantitative analysis software tool provided by the camera’s vendor (GE Volumetrix Mi). The VOI of all bone lesions visible on SPECT findings of patients was determined. The normal control group consisted of normal vertebrae of patients included in this study. The SUVmax of normal vertebrae was determined in spherical VOIs of 20-mm diameter, with no visible metastatic or skeletal degenerative lesion in SPECT or CT images (up to two normal vertebras were included on every patient if there were sufficient normal vertebras available).

### 2.3 Image analysis

According to the corresponding morphologic findings on the CT images of SPECT/CT, bone lesions with abnormal radioactivity concentration were classified as benign, malignant, or undetermined by the consensus of two 10-year experienced nuclear medicine physicians basing on the principle of independent and blinded.

The diagnostic criteria were as follows (adapted from Helyar et al. (18) and Römer et al. (19)): (i) benign lesions showed degenerative changes, such as hyperosteogeny, osteosclerosis, osteophytes, Schmorl’s nodes, bone island, or fracture on CT images. (ii) Undetermined lesions showed the absence of an identifiable anatomic lesion on CT images (referred to as CT occult lesion), or with an identifiable anatomic lesion on CT images, but the diagnosis could not be confirmed. (iii) Malignant lesions revealed osteolytic changes (bone erosion, edge irregularity, no osteosclerosis, or a soft-tissue mass), osteoblastic changes (sclerotic without a soft-tissue mass), or a variable mix of the 2 on CT images.

### 2.4 Statistical analysis

SPSS 22.0 software (IBM SPSS) was used for statistical analysis. All statistical data are presented as mean ± standard deviation (SD). T-test and Mann–Whitney non-parametric test were used for comparison between groups, and receiver operating characteristic (ROC) curve analyses were performed. The diagnostic accuracy of the SUVmax of quantitative SPECT/CT was assessed by calculating the area under the curve (AUC). Cutoff values for optimal sensitivity and specificity have been determined by the ROC curves. *P* < 0.05 was considered statistically significant.

## 3 Results

### 3.1 General clinical characteristics of the participants in Part I

A total of 167 patients were enrolled; 114 patients only had benign lesions, 45 patients only had malignant lesions, and eight patients had benign and malignant lesions. There was no significant difference in age, sex, height, weight, body mass index (BMI), injection dose, and waiting time between benign and malignant patients (P > 0.05) (**Table 1**).

**Table 1:** General clinical characteristics of the participants in Part I.

	Benign	Malignant	Test value	*P* value
Number	122	53		
Male/female	73/49	36/17	χ^2^ = 1.03	0.31
Age (y)	60.63 ± 10.77	57.87 ± 12.74	z = −1.11	0.27
Height (cm)	163.497 ± 9.83	163.323 ± 7.08	z = −0.07	0.95
Weight (kg)	61.38 ± 9.83	61.41 ± 7.88	z = −0.03	0.98
BMI (kg/cm^2^)	22.90 ± 2.84	23.02 ± 2.52	t = −0.16	0.87
Injection dose (MBq)	923.15 ± 70.30	927.22 ± 68.82	t = −0.64	0.52
Waiting time (min)	195.96 ± 20.48	195.77 ± 19.59	z = −0.84	0.40

BMI, body mass index.

### 3.2 The difference of SUVmax between benign and malignant bone lesions and normal vertebrae in Part I and Part II

In Part I, there were a total number of 396 lesions, including 156 malignant (all of them are metastases, including 74 osteoblastic, 73 osteolytic, nine with CT occult lesions) and 240 benign (56 fractures, 151 osteoarthritis, 33 other bone lesions, including fibrous dysplasia of bone and hemangioma). Among the 196 normal vertebrae, 18 were cervical, 105 were thoracic, and 73 were lumbar vertebrae.

The SUVmax of malignant lesions (26.49 ± 12.63) was higher than that of benign lesions (13.92 ± 7.16) and normal vertebrae (6.97 ± 1.52), there were statistically significant in SUVmax among the three groups (*P* = 0.00). There was no statistically significant difference among the SUVmax of osteoblastic (24.86 ± 14.10), osteolytic (25.68 ± 13.52), and CT occult lesions (26.66 ± 12.78) in malignant lesions (*P* = 0.71). Benign lesions were further divided into fractures and non-fracture lesions; the SUVmax of fractures (23.23 ± 14.92) was statistically higher than that of non-fracture lesions (13.55 ± 6.83) (*P* = 0.00), whereas statistically lower than that of malignant lesions (*P* = 0.04).

In Part II, there were a total number of 49 lesions (15 CT occult lesions, 34 identifiable anatomic lesions), including 17 malignant (all of them are metastases, including three osteoblastic, two osteolytic, 12 with CT occult lesions) and 32 benign (3 fractures, 29 other bone lesions). The SUVmax of malignant lesions (23.35 ± 9.35) was higher than that of benign lesions (13.63 ± 14.17). There were statistically significant in SUVmax between the two groups (*P* = 0.00).

### 3.3 Discrimination accuracy of SUVmax for malignant bone lesions compared with benign in Part I and Part II

**Figure 1:**
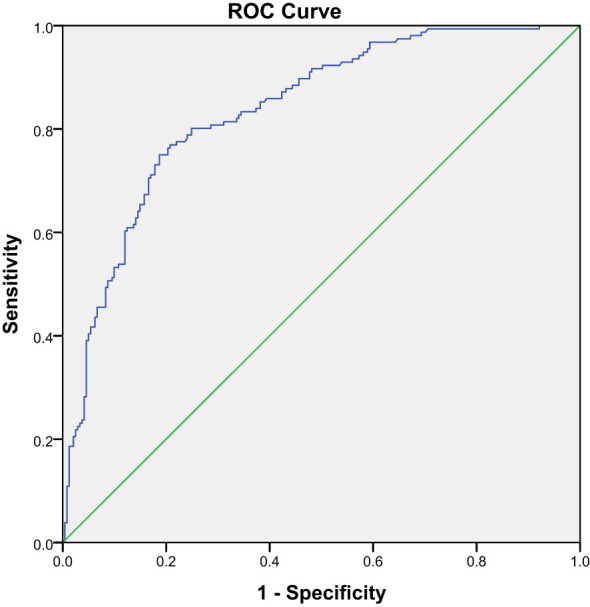
The ROC curve of the SUVmax between malignant and benign lesions in Part I.

In Part I, an ROC curve was drawn to determine the diagnostic accuracy of SUVmax of quantitative SPECT/CT in differentiating between benign and malignant lesions. The ROC curve analysis calculated a high AUC of 0.84 with a 95% CI of 0.80–0.88 (P = 0.00) (**Figure 1**). From the same statistical test, a cutoff value of SUVmax >18.07 was identified as the optimal compromise point between sensitivity and specificity, with values of 75.00% and 81.70%, respectively, in discriminating between benign and malignant lesions.

**Figure 2:**
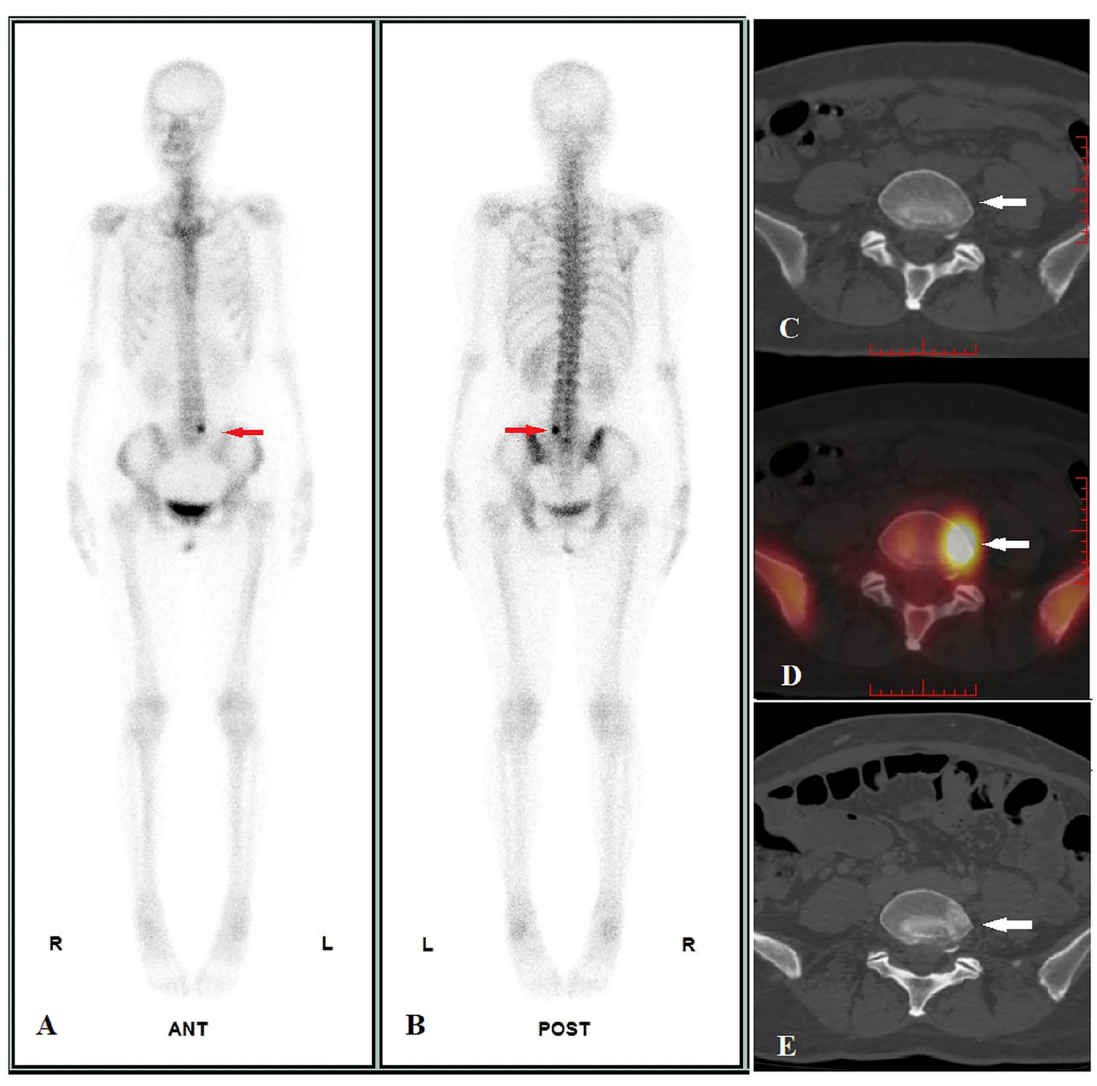
Image display of true positive cases. A 47-year-old female pulmonary neuroendocrine carcinoma patient performed WBS and quantitative SPECT/CT imaging before treatment on 24/06/2021. WBS showed abnormal radioactivity concentration in L5 (**A**, anterior, **B**, posterior, red arrow), and the SUVmax of lesion was 45.41 according to quantitative SPECT/CT (**D**, white arrow), whereas the CT images showed no identifiable anatomic lesion (**C**, white arrow). After 41 days of alectinib therapy, a posttreatment CT scan performed on 14/10/2022 revealed osteoblastic changes in the corresponding area (**E**, white arrow), confirming bone metastasis of L5.

**Figure 3:**
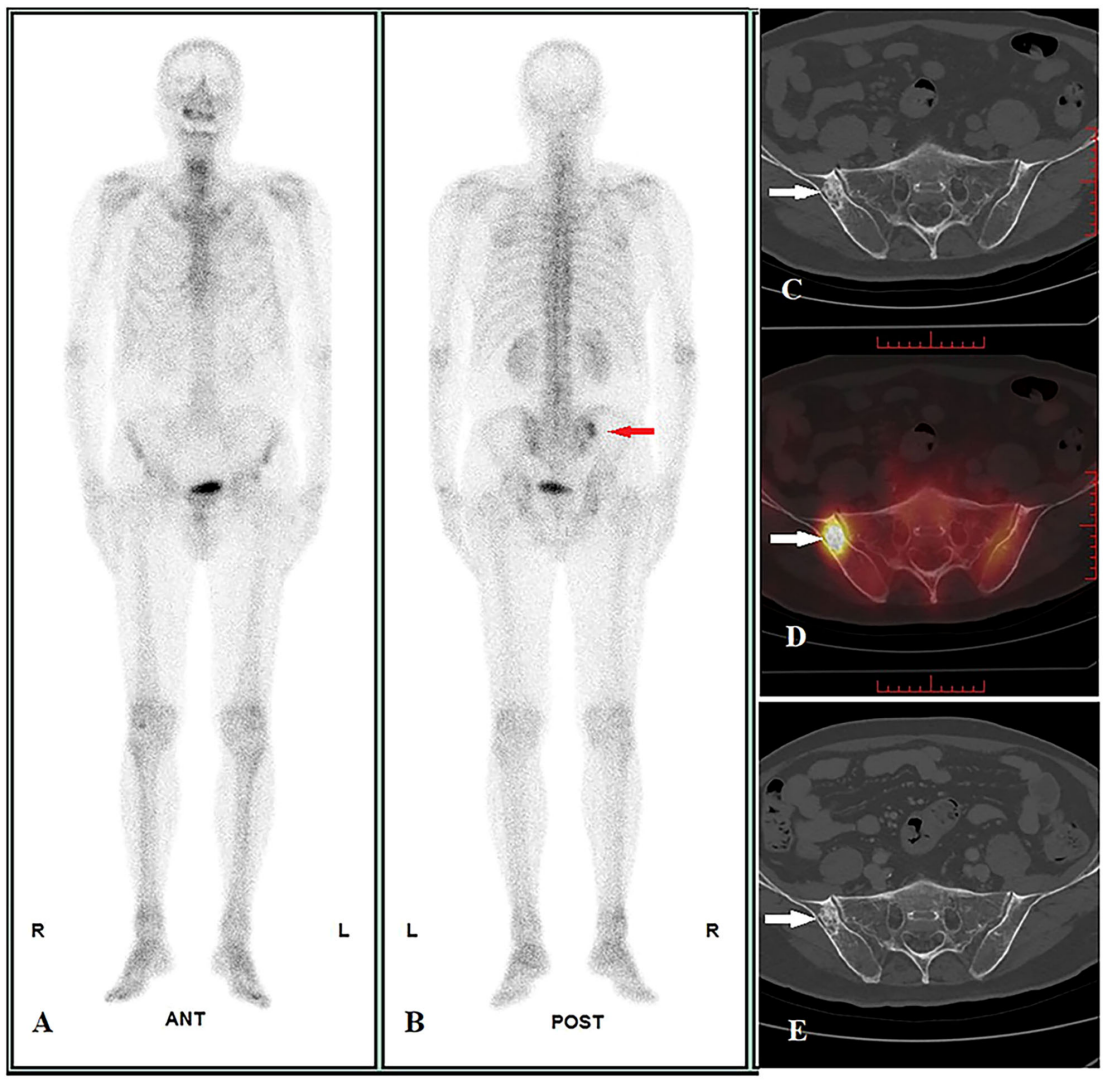
Image display of true negative cases. A 70-year-old male pulmonary adenosquamous carcinoma patient performed WBS and quantitative SPECT/CT imaging before treatment on 07/01/2022. WBS showed abnormal radioactivity concentration in the right ilium at the posterior (**A**: anterior, **B**: posterior, red arrow), and the SUVmax of lesion was 15.60 according to quantitative SPECT/CT (**D**, white arrow); the CT images showed osteoblastic change, but the diagnosis cannot be confirmed (size = 2.6 cm * 1.6 cm, average CT value = 650 HU) (**C**, white arrow). After regional radiotherapy to the chest, systemic chemotherapy, and immunotherapy, a posttreatment CT scan showed new metastasis with the liver and L1 on 14/10/2022 (not shown in the picture). However, at follow-up until 18/03/2023, the size and average CT value of the lesion did not show any significant change on CT images (size = 2.6 cm * 1.6 cm, average CT value = 648HU) (**E**, white arrow), suggesting a benign bone lesion.

In Part II, we used SUVmax ≥18.07 as a diagnostic criterion for malignant bone lesions in 49 solitary undetermined lesions (including 17 malignant and 32 benign lesions). The true-positive malignant lesion was found in 14 lesions (**Figure 2**), true-negative results in 30 lesions (**Figure 3**), false-negative result in 3 lesions, and false-positive result in 2 lesions, and it achieved a high specificity of 93.75% (30/32), a sensitivity of 82.35% (14/17), and an accuracy of 89.80% (44/49) (**Table 2**). The two false-positive lesions were CT occult lesions located in the rib with an SUVmax of 55.74 and 77.93, respectively, which were finally diagnosed as occult fractures according to the follow-up CT imaging. The three false-negative lesions had the SUVmax of 8.43 (rib), 14.67 (ilium), and 15.67 (ilium), respectively. Two lesions located at ilium were CT occult lesion, one lesion located at the rib with identifiable anatomical changes.

**Table 2:** The diagnostic efficacy of SUVmax in solitary undetermined lesions in Part II.

SUVmax	Gold standard		Total
	Malignant	Benign	
Malignant	14	2	16
Benign	3	30	33
Total	17	32	49

## 4 Discussion

With the improvement and clinical application of quantitative SPECT/CT, it has been proved that SUVmax has good repeatability and great potential in the field of bone imaging and can contribute to better patient care and management (10), such as differential diagnosis of benign and malignant bone lesions, and monitor the progression of bone diseases and the response to treatment, especially in the differential diagnosis (20–23).

In Part I of this study, we demonstrated that SUVmax of malignant bone lesions (26.49 ± 12.63) is significantly higher than benign bone lesions (13.92 ± 7.16) and normal vertebrae (6.97 ± 1.52) after excluding the influence of age, sex, height, weight, BMI, injection dose, and waiting time, which can aid in clinical decision-making and improve patient outcomes, and is consistent with previous studies (14, 23, 24). A previous study by Gurkan et al. who used BS for different types of metastases showed no significant difference (p > 0.05) in mean ROImax (ratio of largest lesion to normal bone count on BS) for osteolytic lesions (5.33 ± 3.60), osteoblastic lesions (6.42 ± 4.22), and mixed lesions (6.32 ± 4.03) (25). Similar results were obtained in our study. However, it was in contrast to the study by Li et al. which only included lung cancer patients and showed that SUVmax was significantly higher (P < 0.05) in osteoblastic (29.16 ± 16.63) and mixed (26.62 ± 14.97) lesions than in osteolytic (15.79 ± 5.57) and CT-negative (16.51 ± 6.93) lesions (23). It is possible that the inclusion of different primary tumors contributed to the inconsistent results, and the heterogeneity of different primary tumors should be taken into account in subsequent studies.

Furthermore, an ROC curve analysis was performed to assess the diagnostic accuracy of SUVmax in differentiating benign bone lesions from malignant bone lesions. In our study, the SUVmax cutoff value for distinguishing malignant bone lesions from benign bone lesions was 18.07 and had a high AUC value of 0.84 (95%CI: 0.80–0.88), which is similar to the values registered by Rohani et al. (0.84 vs.0.874), and also with comparable sensitivity (75.00% vs. 73.8%) and specificity (81.70% vs. 85.4%), the differences in the data gathered from the two studies are represented by the cutoff value of ≥18.07 determined by our data and ≥20 from Rohani et al. (21). A previous study also showed a quite similar SUVmax cutoff, with Gherghe et al.’s study that included only breast cancer having an SUVmax threshold of 16.6 with a higher AUC value of 0.974 (95% CI 0.95–0.98) (22). Moreover, the study performed by Lin et al., which included 252 lung cancer bone metastases (mean SUVmax 23.85 ± 14.34) and 140 benign bone lesions (mean SUVmax 9.67 ± 7.47), showed a SUVmax cutoff value of 11.10 for distinguishing bone metastases from osteoarthritic lesions, with an AUC of 0.9097 (95% CI: 0.88–0.94), a sensitivity of 87.70%, and a specificity of 80.71% (23). In general, the SUVmax has preferable diagnostic efficacy in differentiating benign and malignant bone lesions. The reason for the lower SUVmax cutoff value in this study than Rohani et al. may be the lower mean SUVmax of bone metastases in patients with a variety of cancers than in patients only with prostate cancer; the SUVmax of malignant bone lesions in our study was 26.49 ± 12.63, compared with 36.64 ± 24.83 by Rohani et al. Compared with Lin et al. (23) and Gherghe et al. (22), the higher mean SUVmax of benign bone lesions in our data (9.67 ± 7.47 vs. 10.26 ± 4.67 vs.13.92 ± 7.16) was mainly attributed to the higher SUVmax cutoff value, which was due to a high representation of fractures in our study (56 lesions with a high SUVmax of 23.23 ± 14.92) in contrast to only three lesions in the study by Lin et al. and no fracture lesions by Gherghe et al. Of course, the SUVmax in our study differs from other studies, not only because of the different patients and lesions enrolled but also because of some other factors, including instruments, acquisition parameters, reconstruction algorithm, correction technology, and postinjection acquisition time (26).

Moreover, we prospectively used the SUVmax cutoff value of 18.07 as a reference criterion to validate the diagnostic efficacy in 49 solitary undetermined lesions of Part II. To the best of our knowledge, this is the first study to validate the diagnostic efficacy of SUVmax of quantitative SPECT/CT in bone lesions. Our results showed preferable diagnostic efficiency with a sensitivity of 82.35% and a specificity of 93.75%. A significantly higher specificity resulted from validation sets in Part II compared with experimental sets in Part I. Our study provides compelling evidence for using SUVmax of quantitative SPECT/CT imaging as a reliable diagnostic tool for bone lesions.

However, there are still two false-positive lesions and three false-negative lesions. The SUVmax of the two false-positive lesions was 55.74 and 77.93, respectively. There were CT occult lesion and no history of trauma at the rib, which were finally diagnosed as occult fractures according to the follow-up CT imaging. It is well known that fracture (including occult fractures, stress fractures, insufficiency fractures), which could lead to a high MDP concentration due to increased local bone metabolism and perifocal hematoma and necrosis, as well as calcification, is one of the crucial causes of false positives in SPECT/CT (27), and our study’s initial findings revealed that despite the SUVmax of fractures being smaller than that of malignant lesions, with a statistical difference, there is still considerable overlap. In clinical practice, when a solitary undetermined bone lesion with high SUVmax occurs in a vulnerable site (such as ribs), the possibility of fractures (particularly occult fractures and insufficiency fractures) must be excluded by combining a patient history of trauma and short-term imaging follow-up (around 2 weeks later) (28). Another three false-negative lesions had the SUVmax of 8.43 (rib), 14.67 (ilium), and 15.67 (ilium), respectively, and two lesions located at ilium were CT occult lesion, one lesion located at the rib with identifiable anatomical changes, which were finally diagnosed as metastases. The possible reason leading to a low SUVmax was the small volume (<1 cm^3^) of the false-negative lesions. The small volume of the lesion and the low regional bone-blood flow lead to low MDP concentration and low SUVmax; at the same time, due to the partial volume effect, it further leads to lower SUVmax (13), also given the fact that bone uptake of MDP reflects both bone formation and bone turnover, as the overall turnover of bone represents the collective activities of osteoblasts and osteoclasts. Therefore, the possible reason is that the change of bone metabolic activity is in different stages in early CT occult metastasis lesions. It may also be related to the final development of osteoblastic metastasis or osteolytic metastasis, which is related to the dominant position of osteoblastic cell or osteoclastic cells (29). Based on the above, it may also explain why the two CT occult lesions in the ilium presented as false negatives.

The main limitations of our study are as follows: (i) The final diagnosis of bone lesions was mainly based on the gold standard of at least 12 months follow-up, lacking histopathological confirmation. (ii) The study was retrospective, and the data were collected from medical records, which may have introduced some selection bias and limited the scope of the study, such as low proportion of patients with osteophilic tumors such as prostate, breast, and thyroid cancers in the study. (iii) Due to the limited sample size, the heterogeneity of primary tumors was not included in the study and a follow-up project is required. (iiii) Finally, the SUVmax cutoff value used in our study was based on a single-center experience and may not be applied to other institutions or populations. Further studies are needed to validate the optimal SUVmax cutoff value for differentiating malignant from benign bone lesions.

## 5 Conclusions

Our study suggests that the SUVmax of quantitative SPECT/CT is valuable in evaluating solitary undetermined bone lesions. Using a cutoff SUVmax value of 18.07, quantitative SPECT/CT demonstrated high sensitivity, specificity, and accuracy in differentiating malignant from benign bone lesions. Future studies should address these limitations and further investigate the diagnostic utility of quantitative SPECT/CT in more extensive, multicenter cohorts with a broader range of bone lesions.

## Data availability statement

The original contributions presented in the study are included in the article/supplementary material. Further inquiries can be directed to the corresponding author.

## Ethics statement

The studies involving humans were approved by The Ethics Committee of the National Cancer Center/National Clinical Research Center for Cancer/Cancer Hospital and Shenzhen Hospital, Chinese Academy of Medical Sciences and Peking Union Medical College (JS2023-15). The studies were conducted in accordance with the local legislation and institutional requirements. Written informed consent for participation was not required from the participants or the participants’ legal guardians/next of kin in accordance with the national legislation and institutional requirements.

## Author contributions

FD and YL designed the project. XW and YZ analyzed data. ML, TL, and SH contributed to SPECT/CT scans. MZ and RW contributed to image processing. FD wrote the manuscript. YL revised the manuscript. All authors contributed to the article and approved the submitted version.
